# Expression of SARS‐CoV‐2 entry factors in human oral tissue

**DOI:** 10.1111/joa.13391

**Published:** 2021-01-09

**Authors:** Yoshihiko Sawa, Soichiro Ibaragi, Tatsuo Okui, Junro Yamashita, Tetsuro Ikebe, Hiroyuki Harada

**Affiliations:** ^1^ Department of Oral Function & Anatomy Okayama University Graduate School of Medicine, Dentistry, and Pharmaceutical Sciences Okayama Kita‐ku Japan; ^2^ Department of Oral and Maxillofacial Surgery Okayama University Graduate School of Medicine, Dentistry, and Pharmaceutical Sciences Okayama Kita‐ku Japan; ^3^ Department of Oral and Maxillofacial Surgery Okayama University Graduate School of Medicine, Dentistry, and Pharmaceutical Sciences Okayama Kita‐ku Japan; ^4^ Center for Regenerative Medicine Fukuoka Dental College Fukuoka Japan; ^5^ Department of Oral and Maxillofacial Surgery Fukuoka Dental College Fukuoka Japan; ^6^ Department of Oral and Maxillofacial Surgery Tokyo Medical and Dental University Tokyo Japan

**Keywords:** oral tissues, SARS‐CoV‐2

## Abstract

The distribution of cells expressing SARS‐CoV‐2 entry factor angiotensin‐converting enzyme 2 (ACE2) and transmembrane serine protease 2 (TMPRSS2) in human oral tissues were tested. The investigation was conducted with normal flesh tissue and paraffin‐embedded specimens. The ACE2 and TMPRSS2 expression was detected with all subjects in the normal mucosa of the keratinized stratified squamous epithelia of the tongue and non‐keratinized stratified squamous epithelia of the lip and cheek. It was found that ACE2 is expressed in the cytoplasm and on the cell membrane mainly in the stratum granulosum of the epithelia while the TMPRSS2 is strongly expressed on the cell membrane mainly in the stratum granulosum and stratum spinosum, but not in the stratum basale. Antibodies’ reactions for ACE2 and TMPRSS2 were not observed in the nuclei or keratin layer. The expression of ACE2 and TMPRSS2 in the oral epithelia appears to be general, and the expression was also observed in the mucous and serous acini of the labial glands. The SARS‐CoV‐2 may transiently attach to the oral mucosa and the minor salivary glands which are present under all of the oral mucosa. The oral cavity can be considered an important organ for SARS‐CoV‐2 attachment and may provide a preventive medical avenue to guard against COVID‐19 by preventing saliva from scattering.

## Graphical abstract

The ACE2 and TMPRSS2 expression was detected in the tongue, lip and cheek, and observed in the mucous and serous acini of the labial glands. The expression of the ACE2 and TMPRSS2 in the oral cavity appears to be general. The oral cavity can be considered an important organ for the SARS‐CoV‐2 adhesion and may provide a preventive medical avenue against COVID‐19 by preventing saliva from scattering.

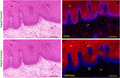

## 1 INTRODUCTION

Taste or olfactory disorders have been reported to occur in a third of patients in the early stages of COVID‐19, the SARS‐CoV‐2 infections (Cevik et al., 2020; Zhang et al., 2020a, 2020b) (International Committee on Taxonomy of Viruses). With the infection, there are patients who suddenly notice taste disorders antecedent to other infection symptoms. In SARS‐CoV‐2 the viral genomic RNA is surrounded by an envelope consisting of a lipid bilayer and outer membrane proteins. The SARS‐CoV‐2 initiates entry into human cells after the Spike protein (S) on the envelope binds to the SARS‐CoV‐2 entry factor angiotensin‐converting enzyme 2 (ACE2) which is a metallopeptidase on the cell membrane. The Spike is cleaved into S1 and S2 by a host cell‐derived protease assumed to be Furin. The S1 binds to ACE2 and S2 is cleaved by another SARS‐CoV‐2 entry factor, a host cell‐derived transmembrane serine protease 2 (TMPRSS2) and membrane fusion proceeds as a result of this. The ACE2 and TMPRSS2 are critical factors in the airway infection for SARS‐CoV‐2 (Sungnak et al., 2020). There are reports that saliva is a source of SARS‐CoV‐2 infection, suggesting that oral tissue including the salivary glands play a role as an organ for viral multiplication in an inapparent infection by SARS‐CoV‐2 (Azzi et al., 2020; Xu et al., 2020a, 2020b) but the presence of the virus receptor has not been shown here. There has also been a detailed report on the rat sialodacryoadenitis virus which is a corona virus infection of the salivary gland (Jacoby et al., 2006). There have been immunohistochemical reports on ACE2 and TMPRSS2 in the oral mucosa, which appear to include reactions to nuclei and keratin (Bertram et al., 2012; Sakaguchi et al., 2020). The expression of ACE2 in oral tissue was reported by a study using the public RNA database (Xu et al., 2020a).

A number of human viral infections of the oral keratinized stratified squamous epithelia are known. Coxsackievirus and adenovirus infect via their receptor in the tight junction of the stratified squamous epithelia (Slots, 2000). Papillomavirus infects the basal cells of the stratified squamous epithelia via wounding or micro trauma (Syrjänen, 2018). Here the virions are produced in the surface of the epithelium and spread into the surrounding tissue, and the viral genome remains as a plasmid in the basal cell nuclei. The production of viral proteins is controlled by differentiation and movement of the infected cells to the mucous surface. Herpes simplex virus type1 infects Langerhans cells and spreads to the oral mucosa from apoptotic Langerhans cells, and the oral cavity is the most important organ for widespread shedding of the virion (Lynch, 2000). The possibility of SARS‐CoV2 proliferation in oral stratified squamous epithelia is still unknown but it may be speculated that virions could attach transiently. The present study aimed to provide details of the distribution of cells expressing the SARS‐CoV‐2 entry factors ACE2 and TMPRSS2 in the cytoplasm and cell membrane in oral tissue at immunohistochemical detection levels.

## 2 MATERIALS AND METHODS

### 2.1 Ethics

The protocol of the experiments for animal use was approved by the Animal Experiment Committee of Fukuoka Dental College (no. 19010). The study for human tissue specimens was approved by institutional review boards of Tokyo Medical and Dental University (No. D2015‐600‐03) and Okayama University (2005‐004). The human study was conducted with written informed consent. All methods were performed in accordance with the relevant guidelines and regulations.

### 2.2 Mouse subjects

An animal study was conducted to establish the distribution of cells expressing ACE2 and TMPRSS2 immunohistochemically. The experimental procedures were prepared following the ARRIVE guidelines. All applicable international, national, and/or institutional guidelines for the care and use of animals were followed. All procedures performed in the studies were in accordance with the ethical standards of the institutional and/or national research committee and with the 1964 Helsinki declaration and its later amendments or comparable ethical standards. All methods were performed in accordance with the relevant guidelines and regulations. We used 4‐week‐old male mice (C57BL/6, *n* = 5) purchased from a commercial vendor (Kyudo, Fukuoka, Japan). Keeping and experiments were performed in a room with a 100% controlled atmosphere which had passed an examination for bacteria and is located in the Fukuoka Dental College Animal Center. Mice were kept healthily under conventional atmosphere conditions with normal feeding in cages. The mice were housed with an inverse 12‐h day‐night cycle with lights on from 7:00 pm in a room where the temperature (22°C) and humidity (55%) were strictly controlled. Immediately after the purchase all the specimens were collected from mice euthanized by induction anesthesia (1 l/min of 2% isoflurane mixed with 30% oxygen and 70% nitrous oxide with an anaesthetic apparatus) followed by intraperitoneal injections with sodium pentobarbital (150 mg/kg, Sumitomo Dainippon Pharma Co., Ltd.) and cervical dislocation.

### 2.3 Human subjects

The investigation was conducted for normal human specimens: oral cells collected by swabs from volunteers including the corresponding author (*n* = 8); oral tissue was surgically dissected from the tongue and cheek of one volunteer (corresponding author); the tissue, was stored at −80°C in a deep freezer, and was surgically removed from patients as well as donations for dissection training were obtained with informed consent. Investigation was also conducted on stored paraffin embedded sections of normal human lip and tongue tissue in samples surgically resected due to cysts and cancer from 20 patients (aged 24–84 years).

### 2.4 Cells

Human epithelial‐like cell lines of oral squamous cell carcinoma HSC2/RCB1945, tongue squamous cell carcinoma HSC3/RCB1975, and tongue squamous cell carcinoma HSC4/RCB1902 were purchased from the Institute of Physical and Chemical Research BioResource Research Center (RIKEN, Tsukuba, Japan). The human glioma cell line LN319 was purchased from Addexbio Technologies. All cells were maintained in minimum essential medium eagle, alpha modification (Sigma‐Aldrich Co. LLC.) with 10% serum.

### 2.5 PCR sampling

Normal tissue of the lingual mucosa from the volunteer, and mouse tissues of the submandibular gland, tongue, small intestine, and kidney were used after the collection according to the method described above. Immediately after excision 5 mm square tissue specimens were ground into a paste with a scalpel on a glass plate in ice and dissolved in the RLT buffer of an RNeasy kit (Qiagen, Inc.). We also used squamous cell carcinoma cell lines HSC2, HSC3, and HSC4. The cells were dissolved in RLT buffer at the 80% confluence in one well of a 6‐well plate. Furthermore, the buccal mucous epithelial total RNA was collected from volunteers. Immediately after grinding a swab (Jonson's cotton bud was best; Jonson & Jonson K.K., Tokyo, Japan) against the volunteer's buccal mucosa eight times at places facing the tongue, the swab was immersed and spun in the RLT buffer of a RNeasy kit (Qiagen) in a 1.5 ml Eppendorf tube on ice, and the swab, swollen with solution was strongly squeezed by sterilized tweezers. The total RNA extraction from tissue and cells was assayed with a QIAshredder column and an RNeasy kit (Qiagen). Contaminating genomic DNA was removed using DNAfree (Ambion) only when many non‐specific bands were found on the gel electrophoresis after PCR.

### 2.6 Microarray

A microarray was used for the HSC3 and LN319. The analysis was outsourced to Cell Innovator Inc. (Fukuoka, Japan). The analysis was performed by 50,600 probes integrated with the human genome DNA microarray 4x44 K v2 (Agilent Technologies Japan, Ltd.).

### 2.7 Real‐time PCR

Reverse transcription was performed on 30 ng of total RNA, followed by 38 cycles (annealin: 55–60°C) of PCR for amplification using the Ex Taq hot start version (Takara Bio Inc.) with 50 pM of primer sets (Table 1) where the specificities had been confirmed by the manufacturer (Thermo Fisher Scientific). The annealing temperature was varied between 55 and 60°C according to the status of the RNA yield and DNA contamination a large number of times. The RT‐PCR products were separated on 2% agarose gel (NuSieve; FMC), visualized by Syber Green (Takara), and the amplified PCR product size was confirmed by gel electrophoresis.

**TABLE 1:** Sequence of primers

RefSeq NM_	mRNA	Front (5'−3')	bp/*T*_m_ (°C)
		Reverse (5'−3')	
007393	Actb	GTTCTACAAATGTGGCTGAGGA	411 59
		ATTGGTCTCAAGTCAGTGTACAG	
001101	ACTB	ATGTTTGAGACCTTCAACAC	495 58
		CACGTCACACTTCATGATGG	
027286	Ace2, transcript^a^ variant 2	CAGTTGAACACAATTCTGAACACCAT	120 59
		TGTCGCCATTATTTCATCCAATCCT	
001371415	ACE2, transcript^a^ variant 1	CCTCCCTGCTCATTTGCTTGG	325 59
		GAAGTCGTCCATTGTCACCTTTGT	
015775	Tmprss2	GTTACTTTGAAGAATGGGATCTGGTG	141 58
		GCTGTTCGCCCTCATTTGCT	
001135099	TMPRSS2, transcript variant 1^a^	CGGCAGGTCATATTGAACATTCCA	318 59
		ACAGTGCTTTCTTAGTCTTTGAGGTG	

Gene symbols are described according to international notation.

^a^ Primer sequences for genes with variants were designed in the region common to variants.

To quantify the mRNA generation, cDNA samples were analyzed by real‐time quantitative PCR. A total of 1 μl of cDNA was amplified in a 25‐μl volume of PowerSYBR Green PCR Master Mix (Applied Biosystems) in a Stratagene Mx3000P real‐time PCR system (Agilent Technologies, Inc.), and the fluorescence was monitored at each cycle. Cycle parameters were 95°C for 15 min to activate Taq followed by 40 cycles of 95°C for 15 s, 58°C for 1 min, and 72°C for 1 min. For the real‐time analysis, two standard curves were created from amplicons for both the β‐actin and target genes in three serial fourfold dilutions of cDNA. The β‐actin or target gene cDNA levels in each sample was quantified against β‐actin or the target gene standard curves by allowing the Mx3000P software to accurately determine each cDNA unit. Finally, target gene cDNA units in each sample were normalized to β‐actin cDNA units. Relative target gene expression units were expressed as arbitrary units, calculated according to the following formula: relative experimental gene expression units =cDNA amounts of experimental samples/cDNA amounts of samples determined as controls. All experiments were repeated at least five times.

### 2.8 Immunostaining

For the cancer tissue specimens, 2‐µm thick paraffin‐embedded sections mounted on 3‐aminopropyltriethoxysilane‐coated slides were used. The sections were deparaffinized in changes of xylene, rehydrated in decreasing concentrations of ethanol, and rinsed with deionized distilled water for 10 min. The slides were immersed in 10 mM sodium citrate buffer (pH 6.0) or Envision Flex Target Retrieval Solution (Agilent Technologies, Inc.), and boiled in a microwave oven for 10 min to break formalin‐formed protein cross‐links masking the antigen. Immunohistochemistry was performed using the avidin‐biotin peroxidase complex technique. After the boiling, the sections were rinsed by 10 mM PBS and then immersed for 10 min in methanol containing 3% hydrogen peroxide, followed by incubation in 0.1% horse serum for 30 min at room temperature. The sections were then incubated with a rabbit anti‐TMPRSS2 (0.5 µg/ml, Abcam Inc., ab92323) and anti‐ACE2 (Abcam, ab15348) diluted into the blocking solution for 8 h at 4°C. After the treatment with primary antibodies the sections were washed three times in PBS for 10 min and immunostained with a horse anti‐ rabbit IgG (0.5 μg/ml, Vector‐Elite ABC kit; Vector Laboratories, Inc.) for 1 h at RT, and then visualized by a Vector VIP substrate kit (Vector) at RT.

Normal human 5–10 mm square tissue specimens of the lingual, labial, and buccal mucosa were obtained from the volunteer by excision with a scalpel under infiltration anesthesia from in the author's laboratory. Mouse tissue of the tongue, small intestine, and kidney were collected after euthanasia by induction anesthesia of the mice with 2% isoflurane (1 l/min) and intraperitoneal injections with sodium pentobarbital (10 ml/kg, Nembutal, Abbott Laboratories). Immediately after excision of tissue, cryo‐embedding in Tissue‐Tek Cryomold (Sakura Finetek Japan Co., Ltd.) was performed by using liquid nitrogen. The frozen 10 μm sections cut in a cryostat on slide glass were fixed in 100% methanol for 5 min at −20°C. The sections were treated with PBS blocking solution containing 0.1% goat serum for 30 min at 20°C and then with a 1 µg/ml rabbit anti‐TMPRSS2 (Abcam) and anti‐ACE2 (Abcam) diluted into the blocking solution for 8 h at 4°C. Double immunostaining was performed on only the mouse kidney sections with anti‐ACE2 (Abcam) and a 1 µg/ml hamster anti‐podoplanin (Biolegend, Inc.) to distinguish renal glomerular podocytes. After the treatment with primary antibodies the sections were washed three times in PBS for 10 min and immunostained for 1 h at 20°C with 0.1 μg/ml of Alexa Fluor 488‐conjugated anti‐hamster IgG and Alexa Fluor 568‐conjugated goat anti‐hamster IgG (Probes Invitrogen Com.). The immunostained sections were mounted in 50% polyvinylpyrrolidone solution and examined by microscope digital camera systems with a CFI Plan Apo Lambda lens series and DS‐Ri2/Qi2 (Nikon Corp.). All experiments were repeated a number of times (5–10) with several sections.

### 2.9 Statistics

All experiments were repeated five times, and the data expressed as mean ±SD. Statistically significant differences (*p* < 0.01) were determined by one‐way ANOVA and the unpaired two‐tailed Student's *t* test with STATVIEW 4.51 software (Abacus Concepts).

## 3 RESULTS

### 3.1 RT‐PCR analysis

**FIGURE 1:**
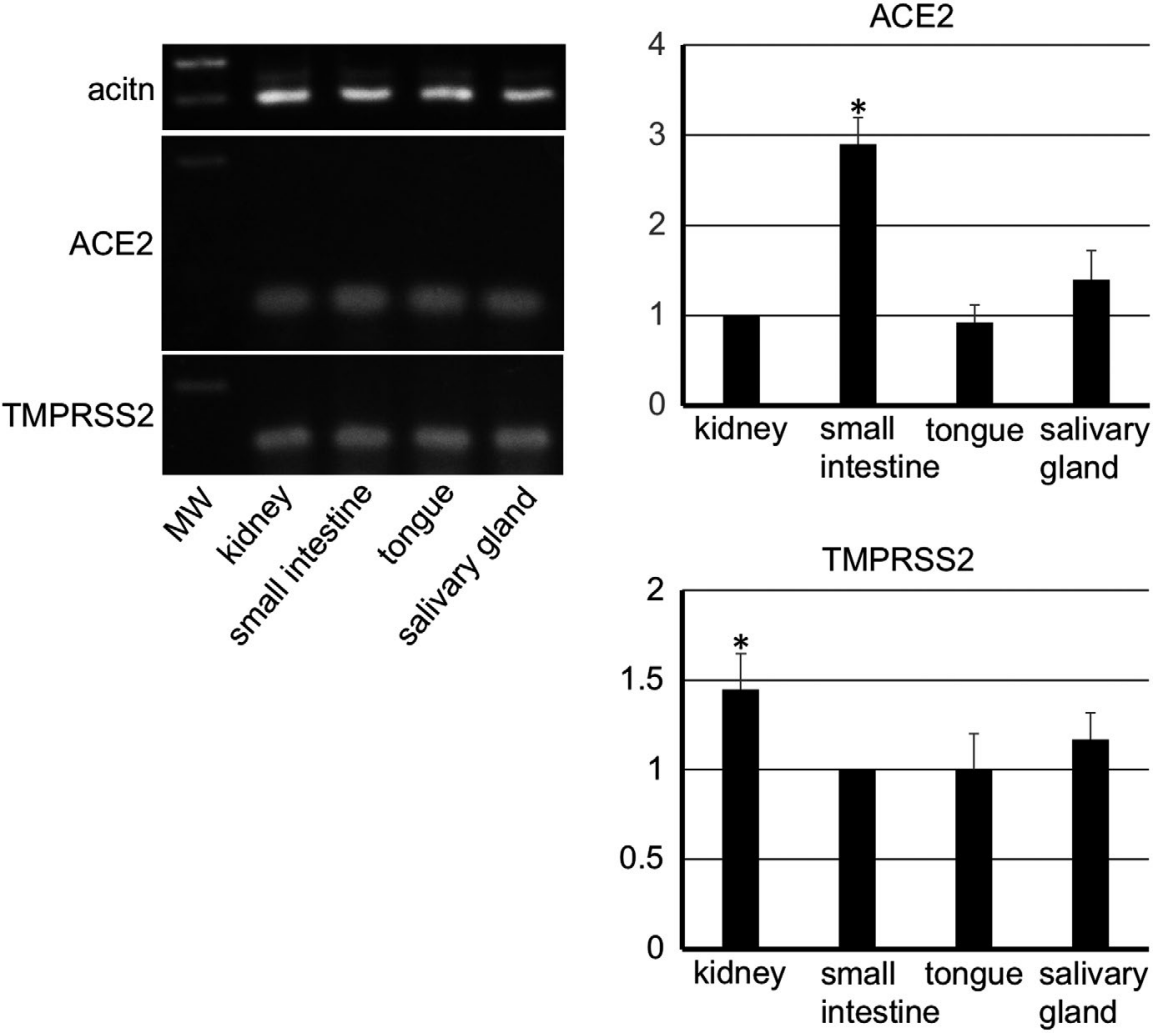
RT‐PCR analysis for mouse tissue. The RT‐PCR analysis for the mouse tissue shows that there are cells expressing ACE2 and TMPRSS2 in the submandibular gland, tongue, small intestine, and kidney. The real time‐PCR analysis shows that the gene expression of ACE2 in the small intestine and TMPRSS2 in the kidney was significantly stronger than in other tissues. Target gene cDNA were normalized to β‐actin cDNA. The relative gene expression was expressed as arbitrary units according to the following formula: cDNA amounts of experimental samples/cDNA amounts of samples determined as controls (ACE2: kidney, TMPRSS2: small intestine). Values are means ± SD. *Significantly different in ANOVA (*p* < 0.01)

**FIGURE 2:**
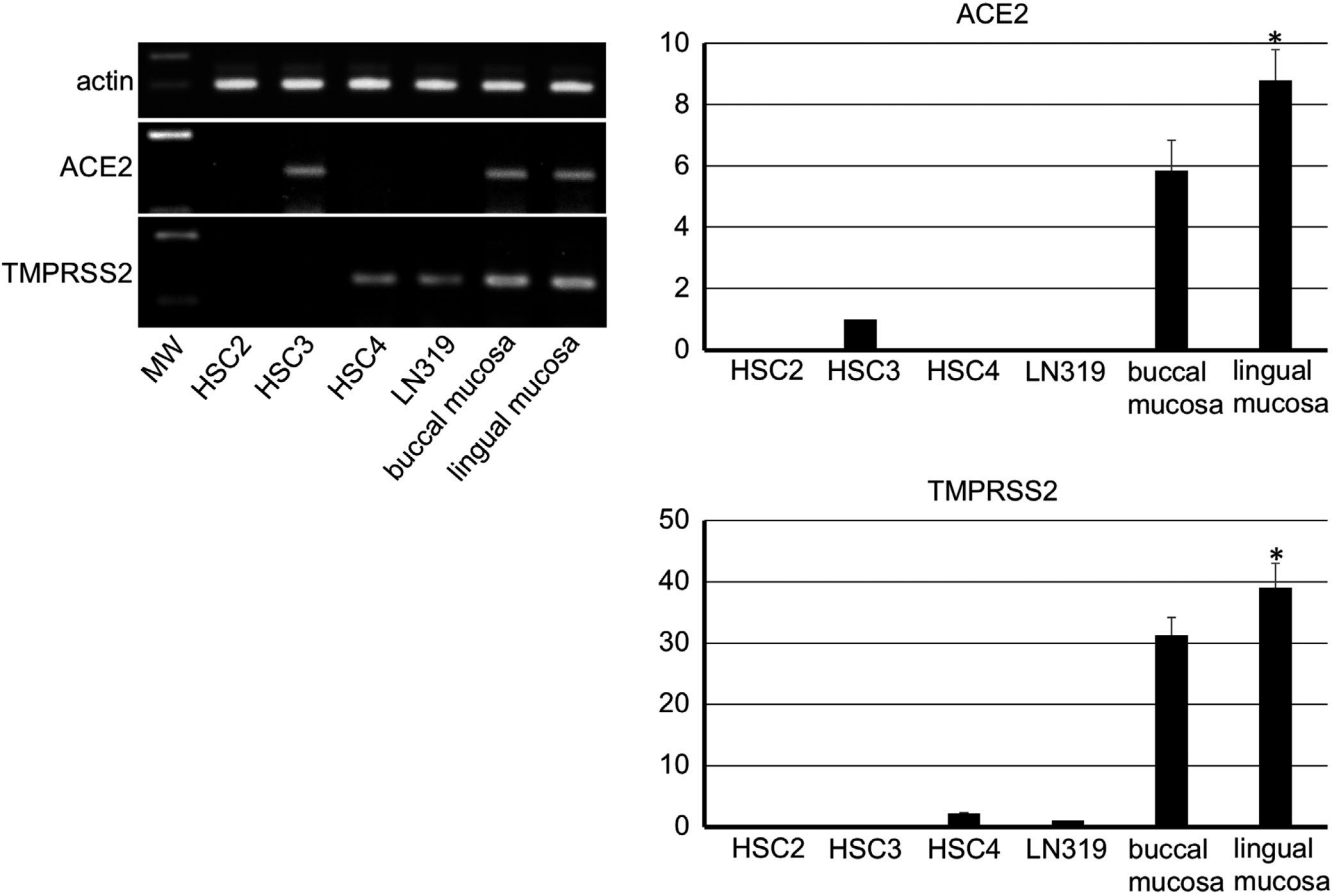
RT‐PCR analysis for human tissue and cells. The RT‐PCR analysis for the human tissue of the lingual mucosa and buccal mucosa including normal keratinocytes shows that there are cells expressing ACE2 and TMPRSS2. In human epithelial‐like oral cancer cell lines, the reaction product of RT‐PCR for ACE2 mRNA was detected in HSC3 but not in HSC2 or HSC4. The reaction product for TMPRSS2 mRNA was detected in HSC4 but not in HSC2 or HSC4. In the human glioma cell line LN319, the reaction product for ACE2 mRNA was not detected while the reaction product for TMPRSS2 mRNA was detected. The real time‐PCR analysis shows that a strong expression of ACE2 and TMPRSS2 genes are present in the lingual and buccal mucosa, and the gene expression of both ACE2 and TMPRSS2 was significantly stronger in the lingual mucosa than in buccal mucosa or cell line controls. Target gene cDNA were normalized to β‐actin cDNA. The relative gene expression was expressed as arbitrary units according to the following formula: cDNA amounts of experimental samples/cDNA amounts of samples determined as controls (ACE2: HSC3, TMPRSS2: LN319). Values are means ± SD of 5 data. *Significantly different in ANOVA (*p* < 0.01)

The mouse tissue specimens from the kidney, small intestine, tongue, and submandibular gland showed expression of the ACE2 and TMPRSS2 genes (Figure 1). The gene expression of ACE2 in the small intestine and TMPRSS2 in the kidney was significantly stronger than in other tissues. In the microarray there was a HSC3 strain with the ACE2 gene expression and a LN319 strain with the TMPRSS2 gene expression (not shown). Therefore, these strains were used as experimental controls in the RT‐PCR analysis of the human tissue. It was observed that in the human epithelial‐like oral squamous cell carcinoma cell lines HSC2, HSC3, and HSC4 gene expression of ACE2 was present in HSC3 while the gene expression of TMPRSS2 was present in HSC4. The LN319 showed that ACE2 was absent but confirmed the presence of TMPRSS2. The normal human specimens from the lingual and buccal mucosa also showed a strong expression of ACE2 and TMPRSS2 (Figure 2). The gene expression of both ACE2 and TMPRSS2 was significantly larger in the lingual mucosa than in other specimens.

### 3.2 Immunostaining of the mouse tissue

**FIGURE 3:**
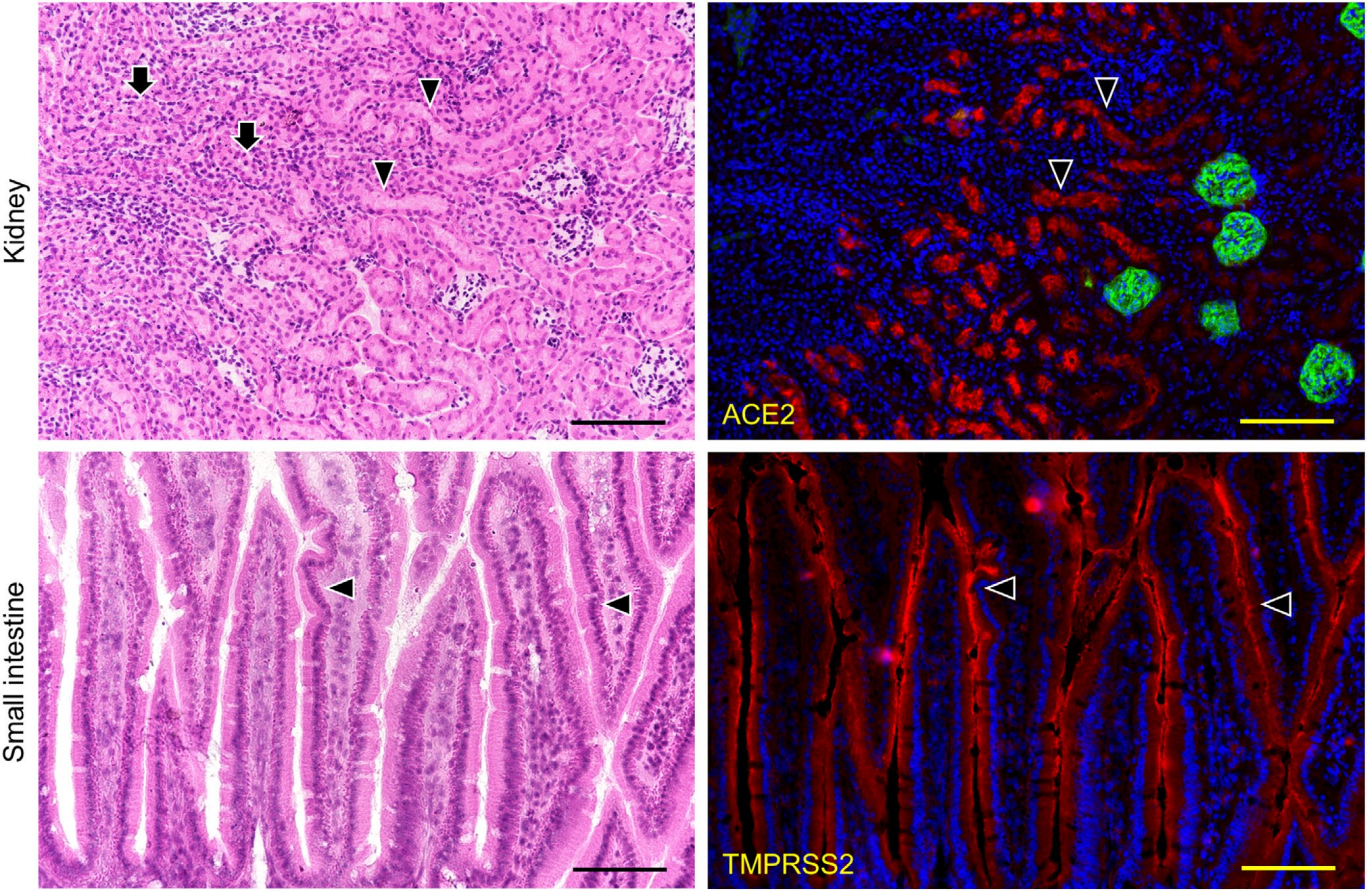
Immunostaining for ACE2 and TMPRSS2 of the mouse kidney and small intestine. HE staining (left panels) and immunostaining (right panels) for ACE2 (red), and for podoplanin (green) with DAPI staining of nuclei (blue). In the mouse kidneys (top panels), the reaction with anti‐ACE2 (arrowheads) was only observed in the proximal tubular cells with brush border (HE), but no other region including the distal tubular cells (HE, arrows) reacted with anti‐ACE2. It is possible to discriminate glomeruli stained with anti‐podoplanin. In the sections of mouse small intestine (bottom panels), the reaction with anti‐TMPRSS2 (red, arrowheads) was only observed in the monolayered columnar epithelial cells of the small intestine (HE), but no other region including the lamina propria mucosae (HE, arrows) reacted with anti‐TMPRSS2. Bar: 100 μm

**FIGURE 4:**
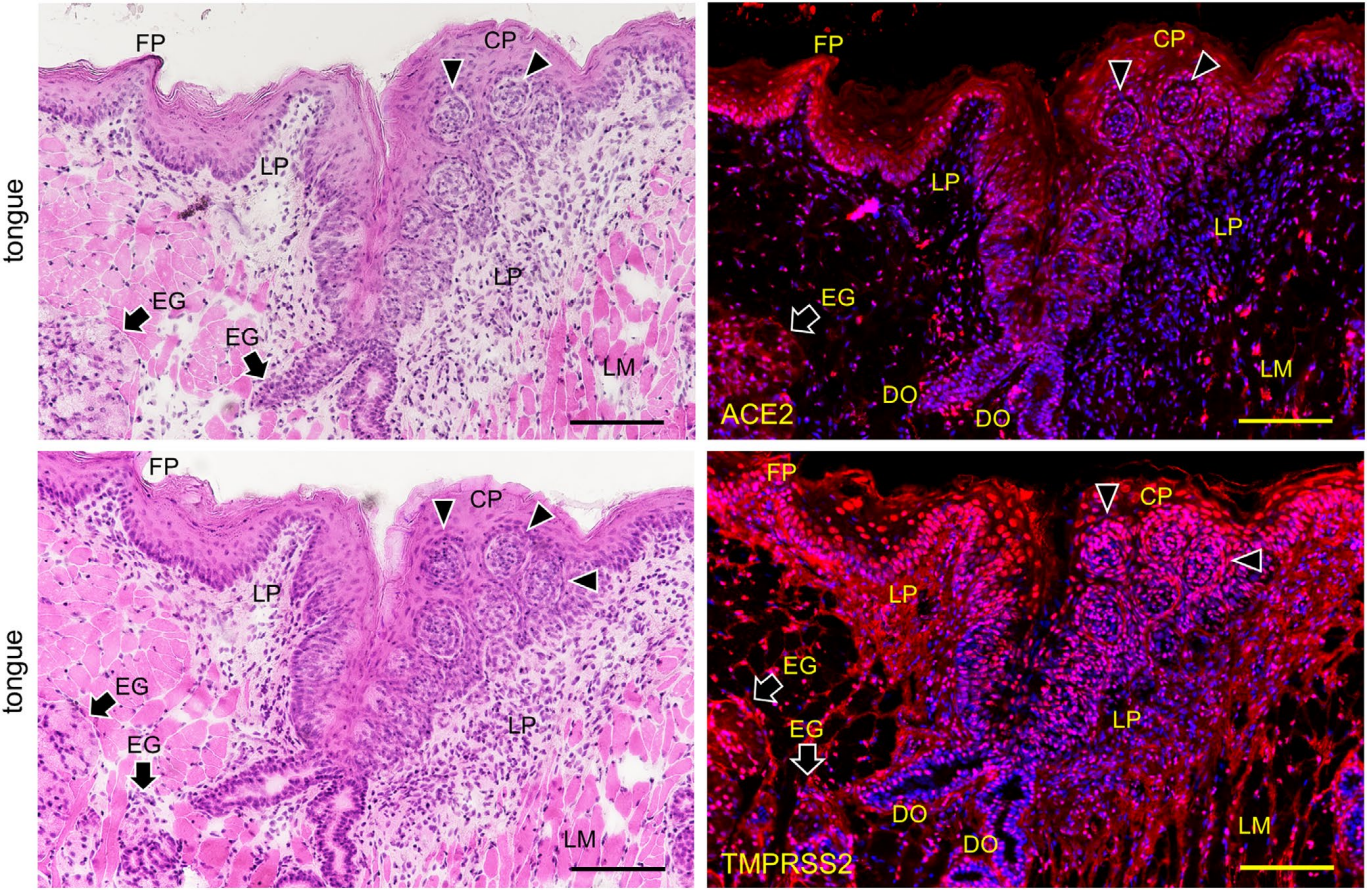
Immunostaining for ACE2 and TMPRSS2 of the body of the mouse tongue. HE staining (left panels) and immunostaining (right panels) for ACE2 (upper right panel, red) and TMPRSS2 (lower right panel, red) with DAPI staining of nuclei (blue). Reactions with anti‐ACE2 and anti‐TMPRSS2 are observed in the keratinized stratified squamous epithelia, containing filiform papilla (FP), but rarely in the cornified layer of the dorsal surface. Reactions with anti‐ACE2 and anti‐TMPRSS2 are observed in the circumvallate papilla (CP). Reactions with anti‐ACE2 and anti‐TMPRSS2 are observed in the pale barrel‐shaped taste buds (arrowheads) of the lateral walls of the furrow encircled circumvallate papilla. Furthermore, reactions with anti‐ACE2 and anti‐TMPRSS2 can be observed in the serous acini of the von Ebner glands (EG) which have duct orifices (DO) connected to the bottom of the furrow. There is reaction with anti‐ACE2 in the blood vessels in the intrinsic lingual muscle but not in the connective tissue of the lamina propria (LP). There is also reaction with anti‐TMPRSS2 in the perimysium of the intrinsic lingual muscle and in the connective tissue of the lamina propria (LP). Bar: 100 μm

**FIGURE 5:**
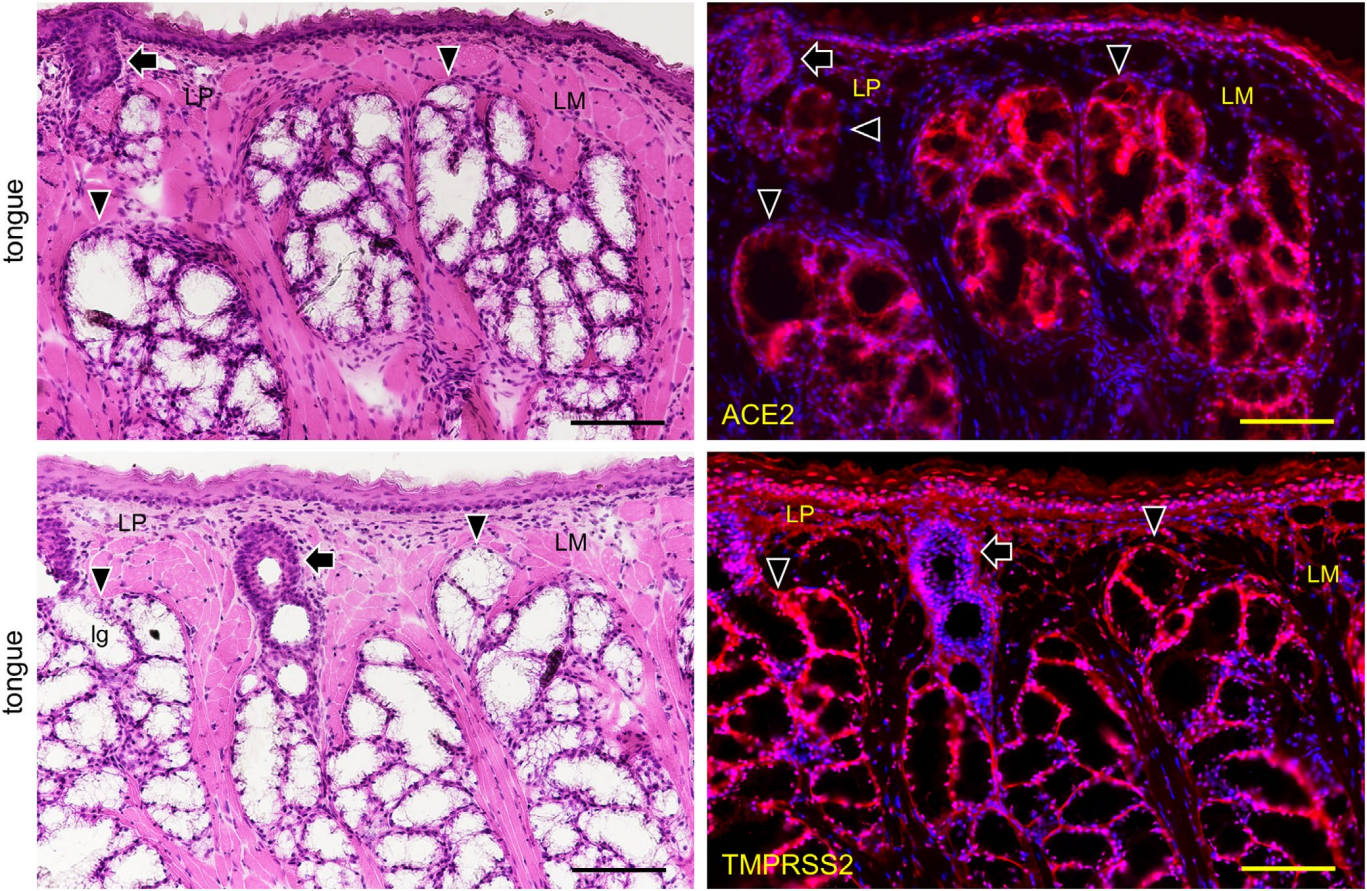
Immunostaining for ACE2 and TMPRSS2 of the root of the mouse tongue sections. HE staining (left panels) and immunostaining (right panels) for ACE2 (upper right panel, red) and TMPRSS2 (lower right panel, red) with DAPI staining of nuclei (blue). Reactions with anti‐ACE2 and anti‐TMPRSS2 are observed in the keratinized stratified squamous epithelia. Reactions with anti‐ACE2 and anti‐TMPRSS22 are observed in the mucous acini of the posterior lingual gland (arrowheads) with the duct also expressing both molecules (arrows). Reaction with anti‐ACE2 is absent in the lingual muscle (LM) but weakly observed in the blood vessels of the lamina propria (LP). Reaction with anti‐TMPRSS2 is absent in the lingual muscle but weakly present in the perimysium and in the lamina propria. Bar: 100 μm

In the kidneys the reaction with anti‐ACE2 was only observed in the proximal tubular cells with brush border and not in other regions including not in glomeruli and distal tubular cells reacted with anti‐ACE2 (Figure 3). In the small intestine the reaction with anti‐TMPRSS2 was only observed in monolayered columnar epithelial cells but not in other regions (Figure 3). Therefore, the tissue was used as experimental controls of RT‐PCR analysis for the mouse tissue. The expression of both ACE2 and TMPRSS2 was present on the keratinized stratified squamous epithelia but was rare in the cornified layer of the dorsal surface of the tongue (Figure 4). The expression of both ACE2 and TMPRSS2 was present on the circumvallate papilla which line up in front of the terminal sulcus on the dorsum of the tongue, and is also present in taste buds, in the serous acini of the von Ebner gland, and in the duct (Figure 4). There was ACE2 expression in the blood vessels in the intrinsic lingual muscle but not in the connective tissue of the lamina propria. The expression of both ACE2 and TMPRSS22 was present in the mucous acini of the posterior lingual gland where the duct also expressed both molecules (Figure 5). The ACE2 expression was absent in the lingual muscle but weakly positive in the blood vessels of the lamina propria. The TMPRSS2 expression was absent in the lingual muscle but present in the perimysium and the lamina propria.

### 3.3 Immunostaining of the normal human tissue

**FIGURE 6:**
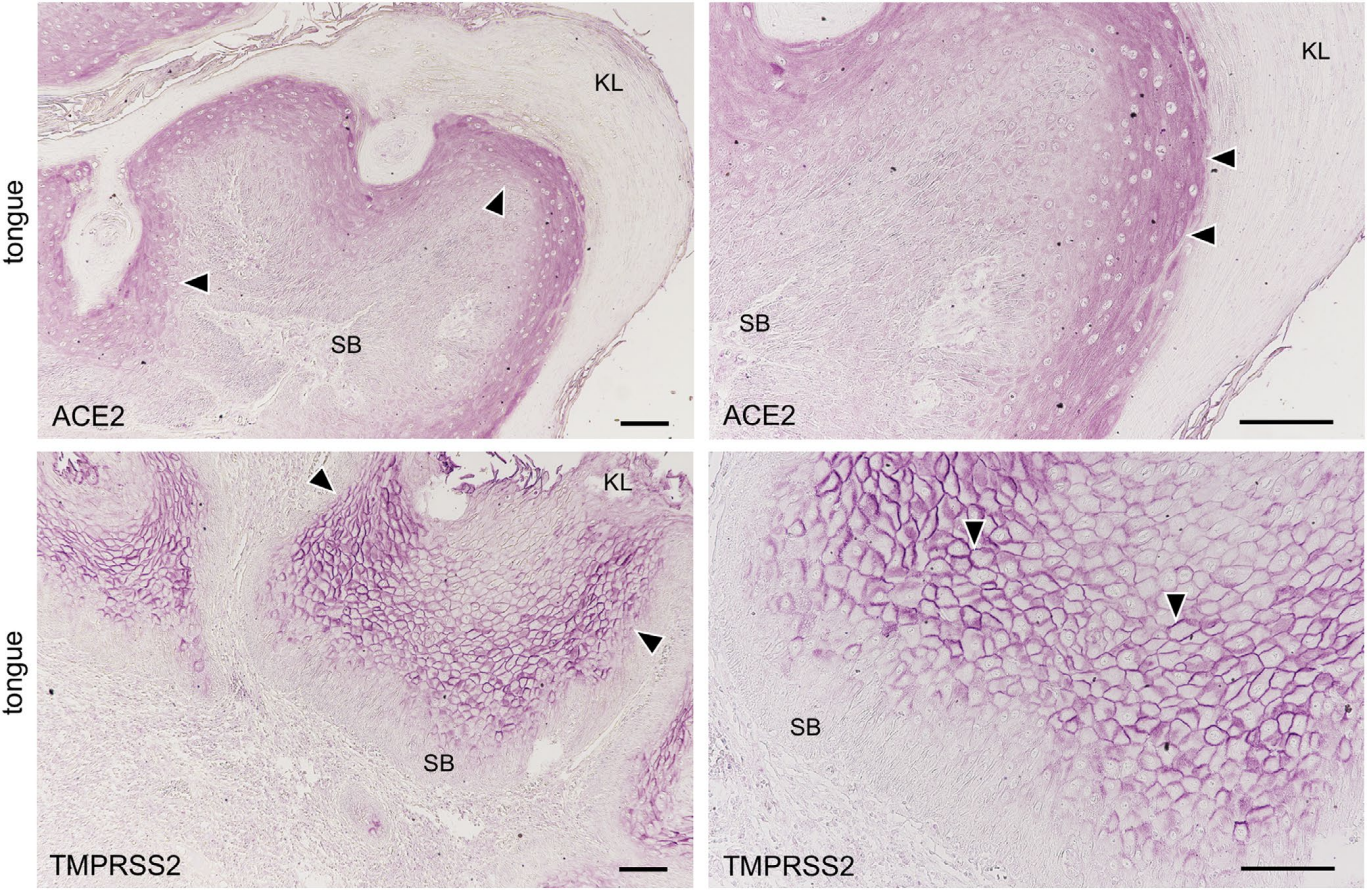
Immunostaining for ACE2 and TMPRSS2 in the paraffin‐embedded normal epithelia. ACE2 (upper panels) and TMPRSS2 (lower panels). Reactions with anti‐ACE2 and anti‐TMPRSS2 are observed on the keratinized stratified squamous epithelia of human tongue. Reaction with anti‐ACE is observed in the cytoplasm and on the cell membrane mainly in the stratum granulosum of epithelia (arrowheads). Strong reactions with anti‐TMPRSS2 are observed on the cell membrane mainly in the stratum granulosum and stratum spinosum (arrowheads), but not in the stratum basale (SB). These reactions are not observed in the nuclei and keratin layers (KL) except for the cross reaction to broken or overlapped parts of the surface. Bar: 100 μm

**FIGURE 7:**
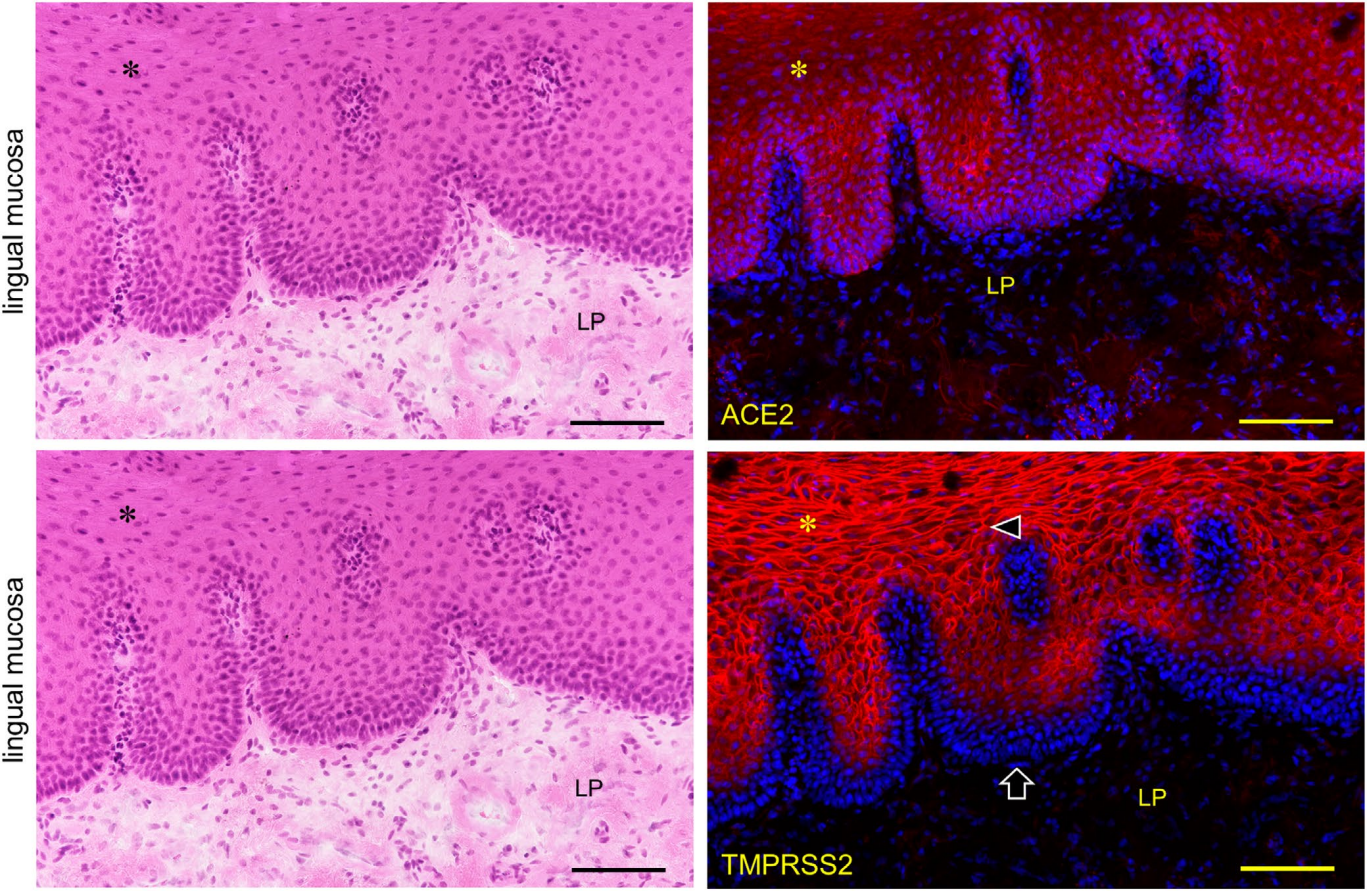
Immunostaining for ACE2 and TMPRSS2 of normal human lingual mucosa. Immunostaining for ACE2 (top right panel, red) and TMPRSS2 (right bottom panel, red) with hematoxylin staining of nuclei (blue). The expression of both ACE2 and TMPRSS2 is observed on the keratinized stratified squamous epithelia (asterisk). The reaction with anti‐ACE is observed in the cytoplasm and on the cell membranes while the reaction with anti‐TMPRSS2 is observed on the cell membranes (arrowheads). There is a very weak reaction with anti‐ACE2 in the lamina propria (LP). TMPRSS2 is not expressed in the stratum basale (arrows) or in the lamina propria. Bar: 100 μm

**FIGURE 8:**
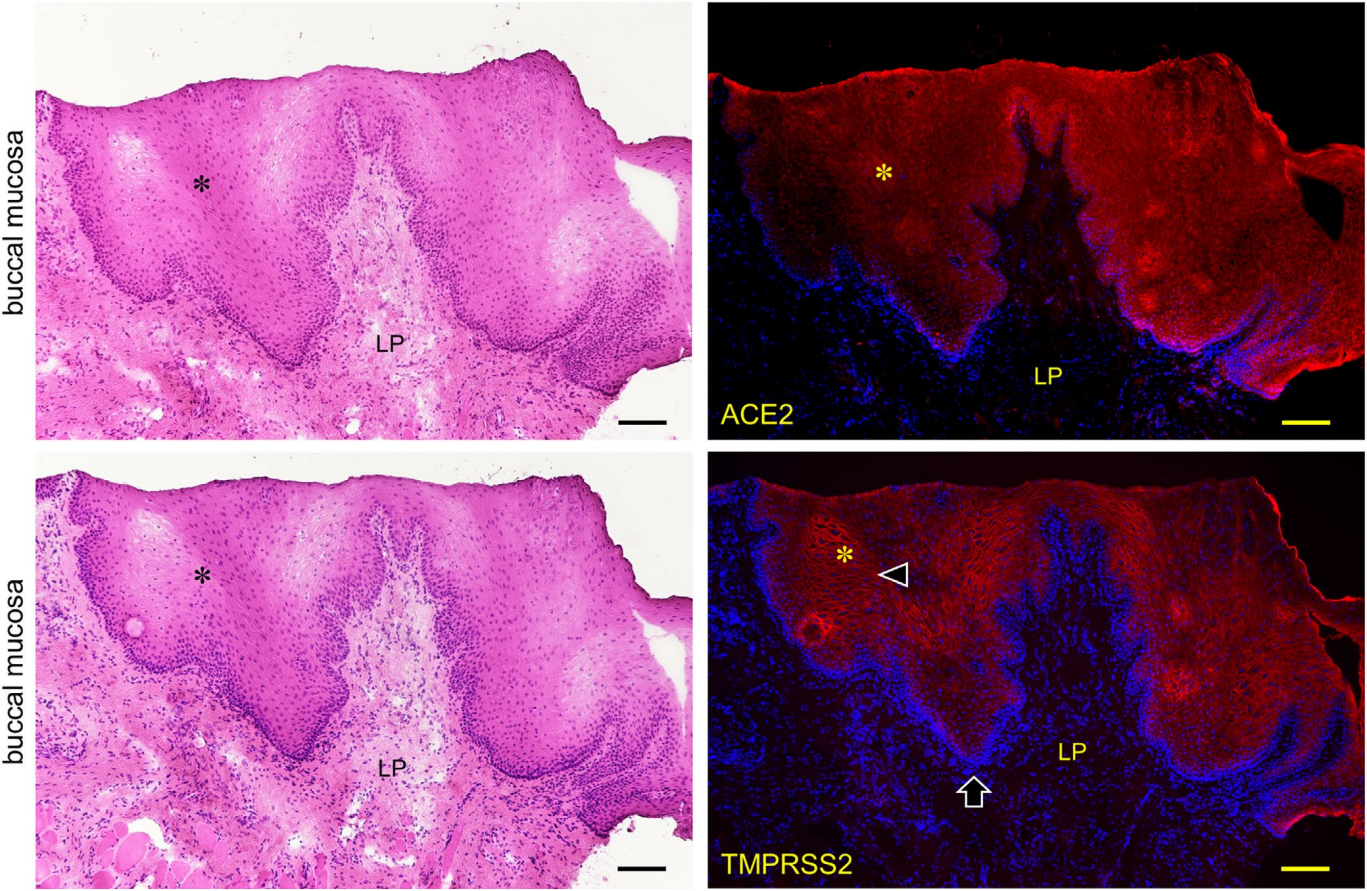
Immunostaining for ACE2 and TMPRSS2 of normal human buccal mucosa. HE staining (left panels) and immunostaining (right panels) for ACE2 (top right panel, red) and TMPRSS2 (bottom right panel, red) with DAPI staining of nuclei (blue). The expression of both ACE2 and TMPRSS2 is observed on the non‐keratinized stratified squamous epithelia (asterisk). Reaction with anti‐ACE is observed in the cytoplasm and on the cell membrane while reaction with anti‐TMPRSS2 is observed on the cell membrane (arrowheads). There is a very weak reaction with anti‐ACE2 in the lamina propria (LP). TMPRSS2 is not expressed in the stratum basale (arrows) or in the lamina propria. Bar: 100 μm

**FIGURE 9:**
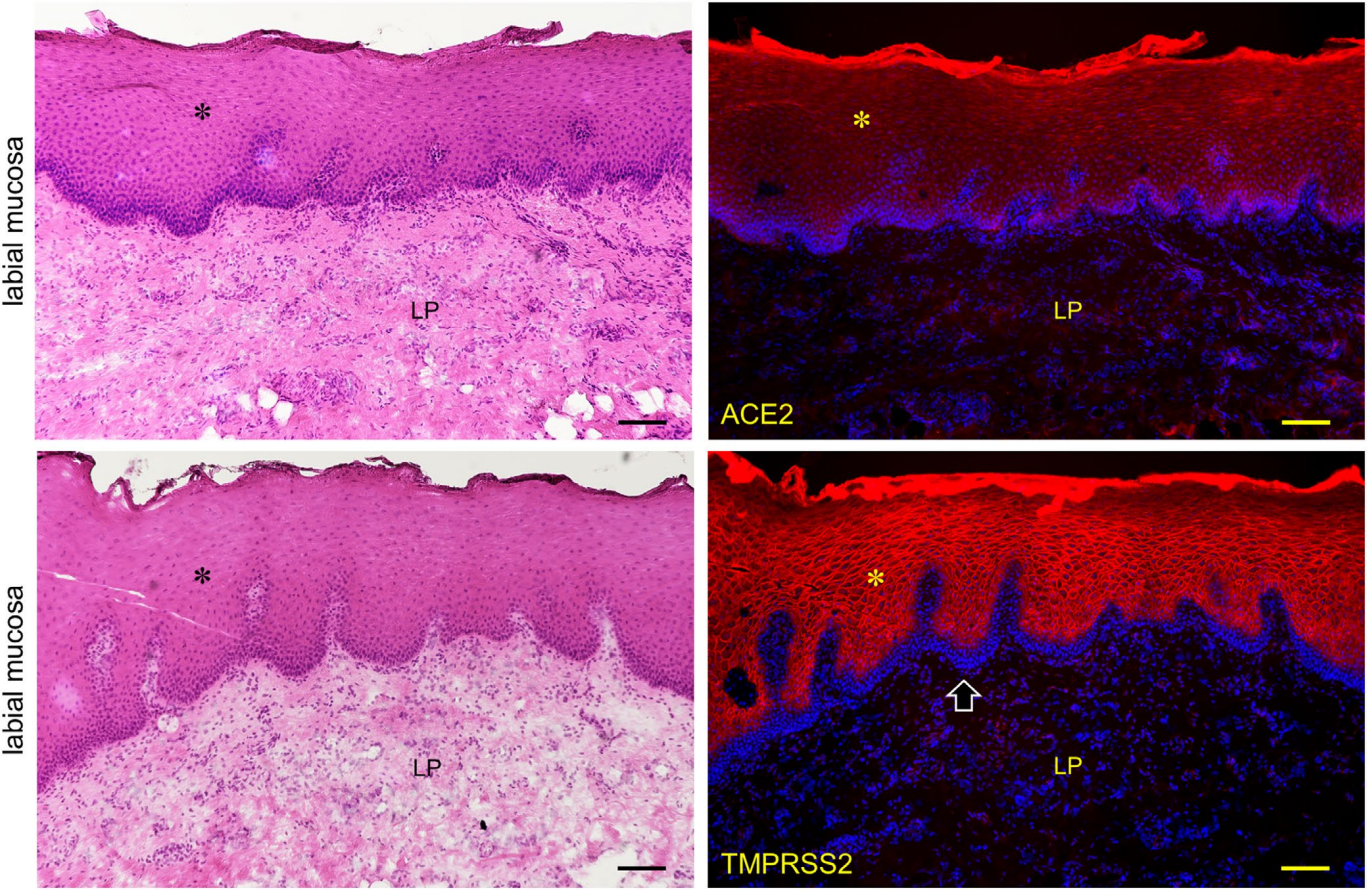
Immunostaining for ACE2 and TMPRSS2 of normal human labial mucosa. HE staining (left panels) and immunostaining (right panels) for ACE2 (top right panel, red) and TMPRSS2 (bottom right panel, red) with DAPI staining of nuclei (blue). The expression of both ACE2 and TMPRSS2 is observed on the non‐keratinized stratified squamous epithelia (asterisk). The reaction with anti‐ACE is observed in the cytoplasm and on the cell membrane while the reaction with anti‐TMPRSS2 is observed on the cell membrane (arrowheads). There is a very weak reaction with anti‐ACE2 in the lamina propria (LP). TMPRSS2 is not expressed in the stratum basale (arrows) or in the lamina propria. Bar: 100 μm

**FIGURE 10:**
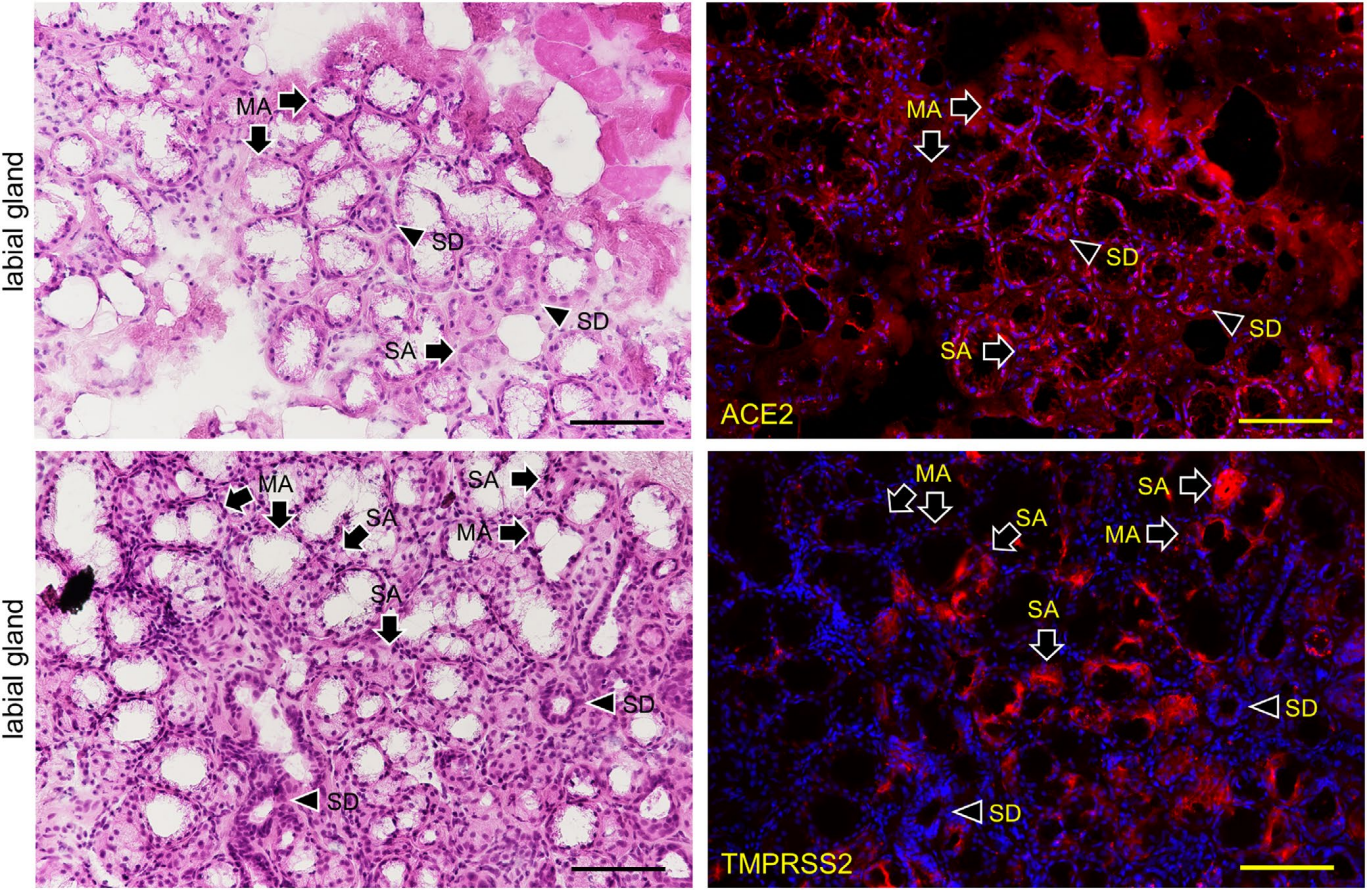
Immunostaining for ACE2 and TMPRSS2 of the labial gland. HE staining (left panels) and immunostaining (right panels) for ACE2 (right upper panel, red) and TMPRSS2 (bottom right panel, red) with DAPI staining of nuclei (blue). The expression of both ACE2 and TMPRSS2 is observed in the mucous acini (arrows, MA) and serous acini (arrows, SA). The expression of TMPRSS2 is stronger in serous acini (arrows, SA) than in mucous acini (arrows, MA). In the striated ducts (SD, arrowheads), the ACE2 is positive but the TMPRSS2 is negative. Bar: 100 μm

The expression of both ACE2 and TMPRSS2 was observed on the keratinized stratified squamous epithelia in the paraffin‐embedded sections (Figure 6). No reactions of antibodies for ACE2 and TMPRSS2 were observed in the nuclei and keratin layer (stratum corneum) except for a cross reaction with the broken or overlapped surface of tissue. It was found that ACE2 is expressed in the cytoplasm and on the cell membrane mainly in the stratum granulosum of the epithelia while TMPRSS2 is strongly expressed on the cell membrane mainly in the stratum granulosum and stratum spinosum, but not in the stratum basale. In the lingual mucosa the expression of both ACE2 and TMPRSS2 was observed in the keratinized stratified squamous epithelia (Figure 7). The expression of ACE2 was present in the cytoplasm and on the cell membrane, and very weakly in the lamina propria. The expression of TMPRSS2 was present on the cell membrane but not in the stratum basale or in the lamina propria. In the buccal mucosa the expression of both ACE2 and TMPRSS2 was observed on the non‐keratinized stratified squamous epithelia (Figure 8). The expression of ACE was observed in the cytoplasm or on the cell membrane while the expression of TMPRSS2 was observed on the cell membrane. There was a very weak expression of ACE2 in the lamina propria. The TMPRSS2 was not expressed in the stratum basale or in the lamina propria. In the labial mucosa the expression of both ACE2 and TMPRSS2 was observed on the non‐keratinized stratified squamous epithelia (Figure 9). The expression of ACE was observed in the cytoplasm and on the cell membrane while the expression of TMPRSS2 was observed on the cell membrane. There was a very weak expression of ACE2 in the lamina propria. The TMPRSS2 was not expressed in the stratum basale or in the lamina propria. In the labial gland the expression of both the ACE2 and TMPRSS2 was observed in the mucous and serous acini (Figure 10). The expression of TMPRSS2 was stronger in the serous acini than in the mucous acini. In the striated ducts the ACE2 was positive but the TMPRSS2 was negative.

## 4 DISCUSSION

### 4.1 Gene expression of ACE2 and TMPRSS2 in mouse organs, human oral tissue, and cancer cell lines

It was shown that ACE2 and TMPRSS2 gene‐expressing cells are present in the mouse submandibular gland and tongue as well as in the small intestine and kidneys (Figure 1). The gene expression of ACE2 in the small intestine and TMPRSS2 in the kidneys was significantly stronger than other tissues. These findings make it plausible that the results of the immunostaining can be considered reliable. Elsewhere, there has also been a report of ACE2 expression in an oral cancer cell line (Hinsley et al., 2017). The highest ACE2 levels were observed in the primary cultured normal oral keratinocytes, with lower levels observed in the head and neck squamous cell carcinoma cell lines SCC4, H357, and Cal27, and ACE2 was not detected in primary cultured normal oral fibroblasts (Hinsley et al., 2017). There have also been reports of TMPRSS2 expression in oral cancer and the cell lines (Cheong et al., 2009; Chodroff et al., 2016). Since the ACE2 gene expression in the tongue squamous cell carcinoma cell line HSC3 and the TMPRSS2 gene expression in the human glioma cell line LN319 were established by a microarray, HSC3 and LN319 were used as experimental controls of the RT‐PCR analysis for the ACE2 and TMPRSS2 gene expression in the human tissue specimens. The ACE2 gene expression was not established in HSC2 or HSC4 by RT‐PCR while it was established in HSC3. The TMPRSS2 gene expression was not established in HSC2 or HSC3 while it was established in HSC4 (Figure 2). It may be postulated that the ability to express ACE2 and TMPRSS2 genes has been deleted in the oral cancer cell lines. The ACE2 and TMPRSS2 mRNAs were detected in both the normal human lingual mucosa collected by excision and the buccal mucous cells collected by grinding with a swab, and the gene expression of both ACE2 and TMPRSS2 was stronger in the lingual and buccal mucosa than in cell line controls, indicating that oral mucous epithelial cells express ACE2 and TMPRSS2 genes, in agreement with previous studies (Cheong et al., 2009; Chodroff et al., 2016; Hinsley et al., 2017).

### 4.2 The expressions of ACE2 and TMPRSS2 proteins in the mouse tissue

Immunohistochemical investigations of the expression of ACE2 protein showed it to be clearly identified in small and large arteries, type II alveolar cells, the stratified squamous epithelium of the esophagus, in the monolayered columnar epithelium of the small intestine, and in the brush border and cytoplasm of proximal tubular cells of the kidneys (Hamming et al., 2004). An immunohistochemical overview of the ACE2 expression at low magnification has also been reported for the oral epithelia (Hamming et al., 2004). In this study the reaction of anti‐ACE2 in the kidneys was confirmed only in the proximal tubules but not in any other region including the glomeruli, and the distal tubules reacted with anti‐ACE2 suggesting that this antibody is useful for the immunohistochemical investigation of ACE2 expression (Figure 3). The TMPRSS2 is a type II transmembrane‐bound serine protease highly localized in the prostate and overexpressed in neoplastic prostate epithelium (Vaarala et al., 2001). The TMPRSS2 was originally reported to be a small intestine associated protease (Paoloni‐Giacobino et al., 1997) and was found to be present in vascular endothelial cells (Aimes et al., 2003), and the gene expression of TMPRSS2 has been investigated in adult mouse and human tissue (Vaarala et al., 2001). Human TMPRSS2 has been detected in the prostate, colon, stomach, and salivary glands by northern blot analysis and in situ hybridization analyses of mouse adult tissue has established that TMPRSS2 was expressed in the epithelia of the gastrointestinal, urogenital, and respiratory tracts (Vaarala et al., 2001). In this study the reaction of anti‐TMPRSS2 in the small intestine was confirmed only in monolayered columnar epithelial cells but in no other region including the lamina propria mucosae did it react with anti‐TMPRSS2, suggesting that this antibody is useful for the immunohistochemical investigation of the TMPRSS2 expression (Figure 3).

In the mouse tongue the expression of both ACE2 and TMPRSS2 was observed on the circumvallate papilla, in taste buds, in the serous acini of the von Ebner gland under the taste buds, and in ducts (Figure 4). The expression of both ACE2 and TMPRSS22 was also observed on the mucous acini of the posterior lingual gland where the ducts also express both molecules (Figure 5). Since the reaction to nuclei was also observed despite blocking, it appears that the antibodies have inherent affinities to nuclear proteins of the mouse oral tissue different from the kidneys and intestines (Figure 3), and are also different from human tissue (Figure 6). Overall, this suggests that the expression of ACE2 and TMPRSS2 is commonly present in vertebrate oral mucosa and salivary gland epithelial cells. It is thought that the taste disorders in the early stages of SARS‐CoV‐2 infection may be involved in the ACE2 and TMPRSS22 expression in the taste buds and Ebner gland (Cevik et al., 2020; Zhang et al., 2020a, 2020b). There is ACE2 expression in blood vessels as also reported elsewhere (Bertram et al., 2012) but not in the mucous lamina propria. As there was reaction with anti‐TMPRSS2 in the mucous lamina propria in the mouse oral tissue but not in the mouse small intestine (Figure 4), the TMPRSS2 protein distribution in the connective tissue may be different in mouse organs.

### 4.3 The expression of ACE2 and TMPRSS2 proteins in human oral tissue

The expression of ACE2 and TMPRSS2 was investigated on paraffin‐embedded tongue specimens. The expression of both ACE2 and TMPRSS2 were confirmed in all subjects (Figure 6). The reactions of antibodies for ACE2 and TMPRSS2 to the oral epithelia were observed in the cytoplasm and on the cell membrane, but not in nuclei and keratin layers, indicating that the antibodies for ACE2 and TMPRSS2 used in this study are useful in the investigation of paraffin‐embedded specimens. It was found that the stratum granulosum expresses both ACE2 and TMPRSS2 on the cell membrane, suggesting that SARS‐CoV‐2 is able to infect human oral keratinized stratified squamous epithelia via wounding or microlaceration like papillomavirus (Syrjänen, 2018). Since the expression of ACE2 and TMPRSS2 is rarely or not observed in stratum basale, it is thought that the production of ACE2 and TMPRSS2 is controlled in the differentiation and movement of basal cells to the mucous surface.

In the lingual mucosa of the normal fresh sample, the expression of both ACE2 and TMPRSS2 was observed on both the keratinized stratified squamous epithelia of the tongue and the non‐keratinized stratified squamous epithelia of the lip and cheek in the same staining pattern among the tissues (Figures 6, 7, 8). There were differences in the expression patterns of the ACE2 and TMPRSS2 in cells. The ACE2 was present in the cytoplasm and on the cell membrane, and very weakly in the lamina propria, while the TMPRSS2 was present on the cell membrane but not in the stratum basale and in the lamina propria (Figures 6, 7, 8). Considering this result of the ACE2 and TMPRSS2 gene expression in the lingual and buccal mucosa by tissue PCR and recent studies for the oral ACE2 taken together, these suggest that the expression of the ACE2 and TMPRSS2 proteins in the human oral epithelia may be assumed to be general. There is no observation of these proteins in the human taste buds in this study, and the taste disorders in the early stages of SARS‐CoV‐2 infection may be involved in the ACE2 and TMPRSS22 expression in the taste buds and Ebner gland (Cevik et al., 2020; Zhang et al., 2020a, 2020b). Since the tissue mRNA extraction by a swab was successful at the same level as the excision, it is thought that sampling by grinding with a swab to the buccal mucosa is effective for the PCR test of the SARS‐CoV‐2 infection. In the labial gland, the expression of both ACE2 and TMPRSS2 was observed in the mucous and serous acini (Figure 9). The expression of TMPRSS2 was stronger in the serous acini than in the mucous acini. In the striated ducts the ACE2 was also positive while the TMPRSS2 was negative. This may suggest that SARS‐CoV‐2 attaches to the oral mucosa and the orifice of the ducts of salivary glands, and also in the minor salivary glands which are present under all of the oral mucosa.

Among seven types of coronavirus that can infect humans, four types account for 15% of the cause of colds and three types are SARS‐CoV identified in 2002, MERS‐CoV in 2012, and new SARS‐CoV‐2 (Zhang et al., 2020a, 2020b). The SARS‐CoV‐2 is a positive‐sense single‐stranded RNA virus and consists of envelope protein, matrix protein, nucleoprotein, and spike protein which binds to ACE2 with high affinity (Wrapp et al., 2020). The SARS‐CoV‐2 binds to ACE2 and the cell surface protease TMPRSS2 cleaves the SARS‐CoV‐2 spike protein, causing the virus to invade the cell (Hoffmann et al., 2020). The upper airway, lungs, and intestinal epithelial cells are expressing ACE2 and TMPRSS, particularly the nasal mucosal epithelial cells strongly express these (Sungnak et al., 2020). Patients with SARS‐CoV‐2 infections develop pharyngitis and runny nose, and excrete virions from the upper airway. When taking preventive measures against the SARS‐CoV‐2 infection, the fact that SARS‐CoV‐2 can infect not only the respiratory system but also the digestive tract should be taken into consideration because SARS‐CoV‐2 has been identified in feces (Wang et al., 2020). Furthermore, SARS‐CoV‐2 is excreted from the nasal cavity and pharynx even in the asymptomatic stage and is able to infect other hosts (Arons et al., 2020). It may be important from a public health point of view that ACE2 and TMPRSS2 which the virus can attach to are usually expressed in the oral cavity as the viral shedding route.

## 5 CONCLUSIONS

The results may suggest that the oral cavity can be considered an important organ for the SARS‐CoV‐2 attachment before infecting the respiratory tract, lungs, and intestines, and may provide a preventive medical avenue against COVID‐19. This would make it possible to hypothesize that facemasks are very effective in controlling COVID‐19 infection by preventing saliva from scattering and sealing in infected persons as well as the skin and clothes, an issue which attention is presently focused on. It is known that sodium lauryl sulfate, an anionic surfactant with protein denaturing potency, is a potent inhibitor of the infectivity of several enveloped viruses through a denaturation of the viral envelope (Piret et al., 2002). Mouthwash containing sodium lauryl sulfate may be effective in preventing SARS‐CoV‐2 infection through the oral cavity.

## INFORMED CONSENT

A human study was conducted to establish the distribution of cells expressing ACE2 and TMPRSS2 immunohistochemically. The study was approved by the Institutional Review Board of Tokyo Medical and Dental University (no. D2015‐600‐03).

## CONFLICT OF INTEREST

The authors have no conflicts of interest to declare.

## AUTHORSHIP

Y.S. designed the study and wrote the paper. S.I., T.O., J.Y., T.I., H.H. acquired data and performed the analysis and statistics. S.I., T.O., J.Y., T.I., H.H. drafted and critically revised the manuscript. All authors approved the final version of the manuscript.

## ETHICAL APPROVAL

The experimental procedures were prepared following the ARRIVE guidelines. All applicable international, national, and/or institutional guidelines for the care and use of animals were followed. All procedures performed in studies involving human participants were in accordance with the ethical standards of the institutional and/or national research committee and with the 1964 Helsinki declaration and its later amendments or comparable ethical standards. The protocol of the experiments for animal use was approved by the Animal Experiment Committee of Fukuoka Dental College (no. 19010). The study for the human tissue specimens was approved by the institutional review boards of Tokyo Medical and Dental University (no. D2015‐600‐03) and Okayama University (2005‐004). A human study was conducted with written informed consent. All methods were performed in accordance with the relevant guidelines and regulations.

## Data Availability

The datasets used and analyzed during the current study are available from the corresponding author on reasonable request.
